# DNA vaccine containing *Flagellin* *A* gene induces significant immune responses against *Helicobacter pylori* infection: An *in vivo* study

**DOI:** 10.22038/ijbms.2021.54415.12227

**Published:** 2021-06

**Authors:** Hossein Ansari, Maryam Tahmasebi-Birgani, Mahdi Bijanzadeh

**Affiliations:** 1 Infectious and Tropical Diseases Research Center, Health Research Institute, Ahvaz Jundishapur University of Medical Sciences, Ahvaz, Iran; 2 Department of Biotechnology, Islamic Azad University of Ahvaz, Ahvaz Branch, Ahvaz, Iran; 3 Department of Medical Genetics, School of Medicine, Ahvaz Jundishapur University of Medical Sciences, Ahvaz, Iran; 4 Cellular and Molecular Research Center, Medical Basic Sciences Research Institute, Ahvaz Jundishapur University of Medical Sciences, Ahvaz, Iran

**Keywords:** DNA vaccine, flaA protein, Flagellin, Helicobacter pylori, Immunomodulation, In vivo

## Abstract

**Objective(s):**

*Helicobacter pylori* is one of the most prevalent human infectious agents that is directly involved in various upper digestive tract diseases. Although antibiotics-based therapy and proton pump inhibitors eradicate the bacteria mostly, their effectiveness has been declined recently due to emergence of antibiotic-resistant strains. Development of a DNA vaccine is a promising approach against bacterial pathogens. Genes encoding motility factors are promising immunogens to develop a DNA vaccine against *H. pylori *infection due to critical role of these genes in bacterial attachment and colonization within the gastric lumen. The present study aimed to synthesize a DNA vaccine construct based on the *Flagellin A *gene* (flaA)*, the predominant flagellin subunit in *H. pylori *flagella.

**Materials and Methods:**

The coding sequence of *flaA* was amplified through PCR and sub-cloned in the pBudCE4.1 vector. The recombinant vector was introduced into the human dermal fibroblast cells, and its potency to express the flaA protein was analyzed using SDS-PAGE. The recombinant construct was intramuscularly (IM) injected into the mice, and the profiles of cytokines and immunoglobulins were measured using ELISA.

**Results:**

It has been found that *flaA* was successfully expressed in cells. Recombinant-vector also increased the serum levels of evaluated cytokines and immunoglobulins in mice.

**Conclusion:**

These findings showed that the pBudCE4.1-*flaA* construct was able to activate the immune responses. This study is the first step towards synthesis of recombinant-construct based on the *flaA* gene. Immunization with such construct may inhibit the *H. pylori*-associated infection; however, further experiments are urgent.

## Introduction

Behind lung, breast, and colorectal cancers, gastric adenocarcinoma is the fourth most common cancer worldwide, and the second cause of cancer-associated deaths, especially in low and middle-income countries (LMIC) like Asia (1). The majority of gastric cancers are etiologically connected with chronic gastritis induced by the stomach-dwelling bacterium, *Helicobacter pylori *(2). The bacterium is a flagellated microaerophilic Gram-negative bacillus affecting half of the humankind (3), approximately. The prevalence of infections varies from 10–60% in Western countries and reaches up to 90% during adulthood in developing countries (4). Besides, studies have shown that mother-to-child transmission mode, is the most frequent cause of *H. pylori *infection, which can usually be acquired in early childhood, particularly in children aged less than 5  (5)*. H. pylori *can survive in the acidic environment of the stomach, colonize and persist in a specific biological niche within the gastric lumen (6). The helical shape of this human pathogen leads to penetration and residence of the bacteria into the stomach lining, under the mucus and it escapes from the host immune system (7, 8). Multiple lines of evidence have revealed association of *H*.* pylori* infections in the pathogenesis of chronic superficial gastritis, gastric ulcers, and gastric cancer (9, 10). Although the combination of proton pump inhibitors and multiple antibiotic therapies is usually used for *H*.* pylori *infections, the rapid emergence of antibiotic-resistant strains, along with some reported side effects, requires the replacement of such an expensive remedy by another therapeutic approach (11). Vaccine development is a promising approach as most of the *H. pylori*-associated virulence factors have been understood (12). Vaccines not only provide long-term protection but also diminish the side effect of generalized administration of antibiotics on beneficial strains of *H. pylori* for human health (10). On the other hand, the pharmaceutical therapy of symptomatic and asymptomatic subjects remains at risk of developing *H. pylori* infection and severe complications, and vaccines may overcome this problem (13). In the past decades, there have been considerable efforts to expand *H. pylori* vaccines based on their crucial virulence factors, including flagellum proteins, vacuolating cytotoxin, cytotoxin-associated antigen, urease, the pathogenicity island, and neutrophil-activating protein even in their recombinant or native forms. These ways confer some degree of protection in the experimental mouse model, although the involved mechanisms almost remain unanswered (14). Nowadays, vector-based vaccines encoding potential *H. pylori* antigens have also been considered, although none of the mentioned strategies have completely protected the host against infection (15). In recent years, vector-based vaccines have been given special attention by researchers rather than the recombinant protein vaccine because DNA vaccines are relatively safe, stable, and induce both cellular and humoral immunity. Besides, these vaccines can be prepared as easily as polyvalent vaccines by inserting DNA that encodes the pathogen’s antigen into a plasmid of bacteria (14). In this way, targeting conserved and essential genes of *H. pylori* to insert in the vector is another issue that could result in identifying the best vaccine against this successful human pathogen (16, 17). According to available studies, flagellum-dependent motilities are essential factors in colonization of *H. pylori *in the host organisms and formation of strong infection (18). Normally, *H. pylori* possess 2-6 polar and sheathed flagella, whose filaments subsist of two types, encoded by *flaA* and *flaB* genes (19). *FlaA* is the dominant flagellin subunit and *flaB* is the lesser subtype and shares considerable amino acid homology, eventually enabling the bacteria to move in their ecological niche performed by the gastric epithelium mucous layer(20). Regarding mentioned documents, the present study aimed to clone and express the *H. pylori*-derived *flaA *coding sequence into the eukaryotic expression system and evaluate the immunomodulatory function of such recombinant construct in an experimental mouse model.

## Materials and Methods

This study was financially (Project code #OG-96116) and ethically (Ethical code IR.AJUMS.REC.1396.268) approved by Infectious and Tropical Diseases Research Center, Health Research Institute, Ahvaz Jundishapur University of Medical Sciences, Ahvaz, Iran.

***Chemicals***

Dulbecco’s modified Eagle’s medium (DMEM) (Gibco, USA), fetal bovine serum (FBS) (Gibco, USA), penicillin and streptomycin (Life Technologies), saline (PBS)solution (Sigma-Aldrich), phenol and chloroform (Sigma-Aldrich), IPTG (isopropyl-β-D-thiogalactoside) (Sigma-Aldrich), and LB broth (Merck). 

***Isolation and identification of H. pylori***

*H. pylori*-positive gastric biopsies were used to extract the bacteria. The specimens were homogenized with a tissue grinder and inoculated onto Columbia agar plates supplemented with 0.5% (w/v) cyclodextrin, 8.0%(v/v) sheep blood, 10 mg/l vancomycin, 5 mg/l trimethoprim, 2500 U/l cefsoludin, and 2.5 mg/l amphotericin B. Then, the plates were incubated at 37 °C under microaerobic conditions (5% O_2_, 10% CO_2_, and 85% N_2_) for 3–5 days. After that, direct gram stain, direct urease, and oxidase tests were performed on each biopsy along with culture. 

***Cell lines and culture condition***

Human dermal fibroblast cells (HDF) were purchased from the National Cell Bank of Iran, Pasteur Institute, and cultured in Dulbecco’s modified Eagle’s medium (DMEM) supplemented with 10% inactivated fetal bovine serum (FBS) (Gibco, USA), 100 U/ml penicillin, and 100 mg/ml streptomycin (Life Technologies). The cells were grown at 37 °C in 5% CO_2 _humidified atmosphere. All cells were trypsinized every 3 days by 0.25 mM Trypsin-EDTA (Gibco, USA).

***Genomic DNA extraction***

Genomic DNA was isolated from *H. pylori* isolates by the phenol-chloroform method as described by Sambrook (21). The extracted DNAs were stored at -20 °C before use. The integrity of isolated DNAs was checked on 1.5% agarose gel electrophoresis. The concentration and purity of the DNA samples were also evaluated at wavelengths 230, 260, and 280 nm using a Nanodrop ND-1000 spectrophotometer (Nanodrop Technologies, Wilmington, DE, USA).

***Vectors and plasmids***

The pTZ57RT easy vector (Thermo Scientific, Lithuania) and pBudCE4.1 (Invitrogen, USA) were used as T/A cloning vector and eukaryotic expression vector, respectively. 

***The amplification of flaA gene using PCR***

The *H. pylori*-associated *flaA* gene coding sequence was retrieved from National Center for Biotechnology Information (https://www.ncbi.nlm.nih.gov). The primers were designed using Gene Runner software, version 3.5, for amplifying the 1545 bp fragment from *the flaA* gene (Table 1). Two restriction sites for *SalI* and *XbaI *included sense and antisense primers, respectively. The PCR reaction was performed in a total volume of 20 µl containing 1 µl of 10X PCR buffer, 2 mM MgCl_2_, 2.5 µmol/l dNTPs, 100 nmol/l of each primer, 100 ng of DNA sample, and 3 units of Pfu DNA polymerase enzyme (Fermentas, Germany). The final volume is adjusted by nuclease-free water. PCR was advanced as initial denaturation at 95 °C for 5 min, followed by 30 cycles, denaturation at 94 °C for 1 min, annealing at 61 °C for 1 min, and extension at 72 °C for 1 min. Basically, a final extension phase was planned at 72 °C for 10 min. Then the PCR products were analyzed on 2% agarose gel electrophoresis, anticipated by ethidium bromide-staining and visualized by UV trans illuminator. For crediting the accuracy of PCR, Sanger sequencing was performed by the PCR primers through Big Dye Terminators (Applied Biosystems 3130 Genetic Analyzer; Applied Biosystems, Foster City, CA, USA).

***Construction of flaA-containing recombinant plasmids***

The *flaA*-amplified product was purified from the gel using *AccuPrep*® Gel Purification (Roche, Germany) and cloned into the pTZ57R/T T/A cloning vector (Thermo Scientific, Lithuania) according to instructions of the manufacturer. The *E. coli DH5α* competent cells were generated using the calcium chloride method as described by Sambrook and Russell (22). Then the competent cells were cultured in LB agar media containing IPTG (isopropyl-β-D-thiogalactoside) (0.1 M), Xgal (20 mg/ml), and ampicillin (100 μg/ml) for screening the recombinant vectors at 37 °C overnight. Then the white colonies were chosen and re-cultured in LB broth media enriched with 100 μg/ml ampicillin. The recombinant vector was purified using Plasmid Mini Extraction Kit (Bioneer, Korea) according to the instructions of the manufacturer. For confirming the accuracy of T/A cloning, a recombinant vector was double digested with salI and *XbaI* restriction enzymes along with *flaA*-specific primers.

***Sub-cloning of the flaA gene into pBudCE4.1 eukaryotic expression vector***

To prepare the pBudCE4.1-*flaA* construct, recombinant vector of pTZ57RT-*flaA* was double digested with *SalI *and *XbaI*. Then *flaA* was purified using *AccuPrep*® Gel Purification (Roche, Germany) and lightly mixed with linearized pBudCE4.1 (digested with the same restriction enzymes) based on the instructions. Ligase enzyme (T4) was used to generate the pBudCE4.1-*flaA* recombinant construct. The *E. coli DH5α* competent cells were transformed with pBudCE4.1-*flaA *and cultured in plates of LB-Zeocine (25 μg/ml). PCR and *SalI*/* XbaI* double digestion was applied to confirm the pBudCE4.1-*flaA* construct. 

***Transfection of HDF cells with a pBudCE4.1-flaA construct***

When the confluency of the cells reached 80–85%, HDF cells were transfected with Lipofectamine 2000 reagent (Invitrogen, USA). One day before transfection, HDF cells were trypsinized and plated in 6-well plates (1 × 10^6^ cells/well). Recombinant pBudCE4.1-*flaA* and Lipofectamine 2000 reagent diluted by Opti-MEM separately. A diluted vector was added to the diluted Lipofectamine in a 1:1 ratio and incubated at room temperature for 5 min. Then lipid-DNA complex was added to the HDF cells in a serum-free DMEM for 6 hr. Then media was replaced with complete fresh media containing 50 µg/ml Zeocin, a selective marker. The potential of the pBudCE4.1-*flaA *constructs to express the recombinant flaA (rflaA) was evaluated 36 hr post-transfection of the recombinant vector. The HDF cells transfected by pBudCE4.1 were considered as transfection control. 

***RNA extraction***

To confirm the efficacy of the vector to express the *flaA* gene, total RNA was isolated from 1×10^5 ^HDF cells using RNX-Plus reagent (Sinaclon, Iran) according to the procedure of the manufacturer. The purity and concentration of the extracted RNA were analyzed by a Nanodrop ND-1000 spectrophotometer (Nanodrop Technologies, Wilmington, DE, USA) at wavelengths 230 nm, 260 nm, and 280 nm. The RNA was recognized pure if the absorbance ratio of 260 nm/280 nm and 260 nm/230 nm was about 2. 

***Complementary DNA (cDNA) synthesis and RT-PCR***

cDNA was synthesized in a total volume of 20 μl in a reaction containing 100 mM dNTPs, 500 ng/µl RNA, 10 µl 5x RT–PCR buffer, 200 mM oligdT primer, and 1 U/µl M-MuLV reverse transcriptase (Invitrogen, USA). The mixture was incubated for one hour at 42 °C, followed by enzyme inactivation and 15 min at 70 °C. The *flaA* transcript was amplified using the specific primers indicated in Table 1. The reaction was carried out in a total volume of 20 μl containing 50 mM MgCl_2_, 0.25 mM of each dNTP, 2 pmol/l of each primer, 20 ng/µl cDNA templates, and 1U of Taq DNA polymerase (Fermentas, Germany). The final volume was modified with nuclease-free water. PCR was developed in a thermocycler and contained 5 min initial denaturation at 95 °C, then 30 cycles of denaturation; 94 °C for 1 min, annealing; 61 °C for 1 min, extension; 72 °C for 45 sec, final extension; 72 °C for 2 min. In all reactions, a sample without a DNA template (NTC) was considered a negative control. Then PCR products were analyzed on 2% agarose gel electrophoresis dyed with 2% ethidium bromide, and their bands were visualized using ultraviolet light.

***Sodium Dodecyl Sulfate Polyacrylamide Gel Electrophoresis (SDS-PAGE) assay***

48 hr post-transfection, the cells were harvested and centrifuged at 6000 rpm for 20 min at 4 °C. The pallet of the cell was broken ultrasonically (300V, 5s×3), and cell lysate was electrophoresed on SDS-PAGE to trace the rflaA protein regarding its molecular weight (54 kDa). A protein lysate from HDF cells was transfected with an empty pBudCE4.1 vector that was also considered as SDS-PAGE control.

***Immunogenicity assays***

*Experimental animals*

Thirty 6–8 week-old female BALB/c mice with 16–18 g weight were acquired from the animal center of Ahvaz Jundishapur University of Medical Science (Ahvaz, Iran). All animals were housed and maintained in a 23 °C, 50% relative humidity, and 12 hr light–dark cycle consistent with the advice for the care and use of laboratory animals. Mice were allowed to adapt to the laboratory for 1 week before experiments. All studies and projects’ steps were revised and accepted by the institutional animal care and use committee of Ahvaz Jundishapur University of Medical Sciences. Mice were given filtered water and sterilized diet and were divided into three groups randomly. Ten mice were assigned to each group and checked daily. 

*Immunization design with DNA vaccine*

Three groups of mice were defined during *in* *vivo* experiments. These included mice who received *pBudCE4.1-flaA*, pBudCE4.1 vector, and PBS. For immunization, *pBudCE4.1-flaA* recombinant construct was diluted in sterile saline equal to 0.25 μg/μl of DNA vaccine/injection or an equal volume of vector and PBS, administered intramuscularly (7) to the animals every 1 week through 4 injections (day 0, day 7, day 14, and day 28). The immunization resting period between injections 3 and 4 was considered 2 weeks, as proposed by Wang and Lu (23). After four injections, mice received 100 μg of *pBudCE4.1-flaA* in total. All solutions were prepared fresh before each administration. One week after each injection (day 7, day 14, day 21, and day 35), 0.5 ml blood was collected from the mouse tail vein into the tubes, centrifuged at 20 °C at 850 ×g for 20 min, and the supernatant (plasma) was collected and stored at -70 °C until use. 

*Enzyme-Linked Immunosorbent Assay (ELISA)*

Total IgM-, IgG-, IL-2-, IL-4-, IL-12- and INF-γ –ELISA (BosterBio, USA) was performed on immunized mouse serum collected at days 0, 7, 14, 21, and 35 based on the user protocols. 

***Statistical analysis***

Data were entered into Prism® 6 software (GraphPad Software, Inc, La Jolla, CA, USA) and analyzed using one-way ANOVA and Newman–Keuls multiple comparison test or Student’s t-test. The difference between the two groups was statistically considered as significant when the *P*-value was less than 0.05. 

## Results

***Recombinant constructs pTZ57RT-flaA and pBudCE4.1-flaA were validated using PCR***

The existence of the 1545 bp band confirmed that *flaA *had been truly cloned into the pTZ57RT vector. The accuracy of the cloning was also checked by *SalI* and *XbaI *double digestion. The presence of 2886 bp and 1545 bp bands was connected with pTZ57RT and *flaA *amplicon, respectively. The un-cut recombinant construct was used as control, and presence of the 4431 bp band (2886 bp+1545 bp) confirmed that *flaA* was successfully T/A cloned. Similarly, the result of colony PCR and *SalI*/*XbaI* double digestion determined that *flaA* had been successfully sub-cloned in the pBudCE4.1 expression vector. The presence of 4595 bp and 1545 bp bands was connected with pBudCE4.1 and *flaA *amplicon, respectively. Similarly, the un-cut recombinant vector was used as control, and the presence of 6140bp band (4595 bp+1545 bp) confirmed that *flaA* was successfully introduced in the pBudCE4.1 vector (**Supplementary Figures 1–3**). 

***Transfected HDF cells expressed the flaA transcript in RT reaction***

To address this question of whether the *pBudCE4.1*-*flaA *is capable of expressing flaA in HDF cells, RNA was isolated from transfected HDF cells and subjected to RT-PCR using flaA-specific primers. The existence of the 1545bp band confirmed that the cells had uptaking and expressed the construct in an acceptable manner (**Supplementary Figure 4**). 

***The recombinant construct pBudCE4.1-flaA efficiently expressed the flaA protein in HDF cells***

**Figure 1:**
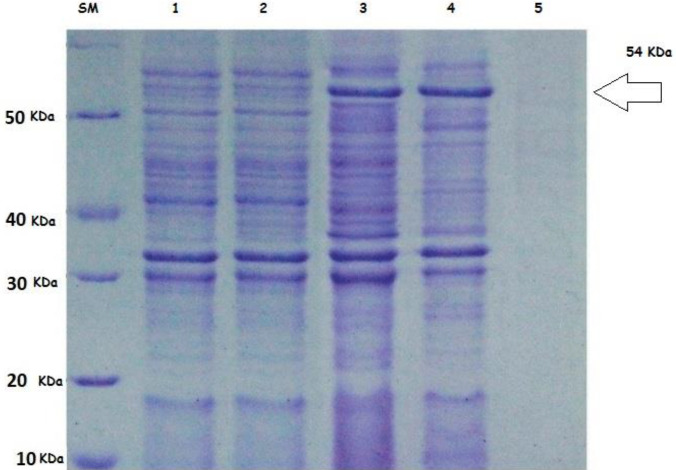
SDS-PAGE analysis of *Helicobacter pylori *flaA protein. SDS-PAGE was used to analyze the expression of rflaA. Recombinant vectors pBudCE4.1–*flaA* and pBudCE4.1 were transfected into HDF. All samples were analyzed by SDS-PAGE, and the protein was stained with Coomassie blue in the gel. Lane SM: molecular mass marker in 10 kDa; Lane 1: whole cell lysate of the *HDF* with pBudCE4.1; Lane 2: HDF including pBudCE4.1–*flaA*, 6 hr after transfection; Lanes 3–4, the whole cell lysate of HDF contained recombinant pBudCE4.1–*flaA*, after 48 hr; Lane 5: Blank control

The presence of flaA protein in transfected HDF cells was examined using SDS-PAGE. Briefly, the HDF cells were transfected with a recombinant vector for 48 hr. Then cell extract was evaluated by SDS-PAGE to find the *flaA* protein. A 54 kDa band was observed on the PAGE, which was identical to the predicted molecular weight of *flaA* (Figure 1). 

***Humoral and cellular responses to recombinant construct pBudCE4.1-flaA***

To evaluate the impact of **the** pBudCE4.1-*flaA* vector on humoral and cellular immune responses, the total serum concentration of IgG and IgM, cytokines IL-2, IL-4, IL-12, and INF-γ were measured during ELISA assay at a specific interval, which has been addressed below.

***pBudCE4.1-flaA significantly induced the levels of IgM and IgG***

**Figure 2:**
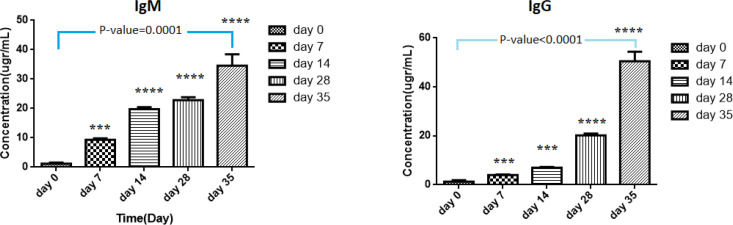
Increased levels of IgM and IgG following injection of pBudCE4.1-*flaA *recombinant construct through four booster injections to mice model of experimental

As illustrated in Table 2, following the injection of 100 μg of *pBudCE4.1-flaA*, the level of IgM was significantly induced in a time-dependent model and increased from 1.20±0.42 μg/ml at day 0 (before immunization) to 34.57±3.89 at day 35 of injection (*P-*value =10-4). The empty vector or PBS has also increased the level of IgM in a time-dependent manner; however, these values were significantly less than the corresponding values for mice receiving recombinant construct (*P-*valu <0.05) (Table 2). The serum level of IgG also significantly increased following each injection and elevated from 1.34±0.54 at day 0 to 50.51±3.91 at day 35 (*P-*value<10^-4^). As observed for IgM, the induction of IgG following the injection of empty vector or PBS was significantly less than in the mice receiving the pBudCE4.1-*flaA* construct (Table 2). As expected, IgM was induced and appeared in the serum earlier than IgG and, this difference remained until day 14 (*P-*value<10^-4^). However, after the third and fourth booster injections, the serum level of IgG was significantly more than the detected level of IgM (*P-*value=0.0075) (Figure 2).

***pBudCE4.1-flaA significantly induced the levels of INF-γ and IL-2, IL-4, IL-12***

**Figure 3:**
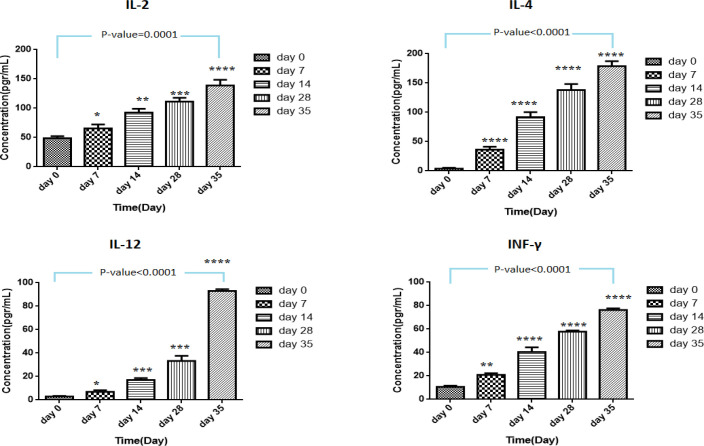
Increased level of IL-2, IL-4, IL-12 and IFN-γ following the injection of pBudCE4.1-flaA recombinant construct through four times booster injections to mice model of experimental

Table 2, summarizes the serum levels of INF-γ, IL-2, IL-4, and IL-12 following administration of 100 μg pBudCE4.1-*flaA*, pBudCE4.1, and PBS through four IM injections. The serum levels of all cytokines increased in a time-dependent aspect as the number of booster injections increased. The concentration of IL-4 was more than the other cytokines on day 35 following pBudCE4.1-*flaA *injection (178.75±8.54 pg/ml), although it appeared in the serum after IL-2 (*P-*value<10^-4^) (Figure 3). On day 35, the serum levels of INF-γ, IL-2, IL-4, and IL-12 reached 138.75±9, 178.75±8.54, 93±1.65, and 76.29±1.42, respectively, which were statistically significant in comparison with corresponding levels in control mice (*P-*value=10^-4^). In fact, the observed immunity by pBudCE4.1 alone and PBS was significantly less than that by recombinant pBudCE4.1-*flaA *construct (*P-*value=10^-4^).

**Table 1:** Primers used for amplification of *flaA* coding sequence from *Helicobacter pylori*

**amplicon size (bp)**	**Sequence**	**Primers**
1545 bp	5’ ACGGTCGACATGGCTTTTCAGGTCAATACAA3’	**Forward primer-** ***SalI***
	5’ CTGTCTAGACTAAGTTAAAAGCCTTAAGATA3’	**Reverse primer-** ***XbaI***

**Table 2:** Serum levels of cytokines IL-2, IL-4, IL-12, and INF—γ and immunoglobulins IgM and IgG, following the four booster injections of pBudCE4.1-flaA, pBudCE4.1, and PBS to an experimental mice model . Data presented as Mean±SD

**pBudCE4.1-flaA**					**Cytokines**
**Day 35**	**Day 28**	**Day 14**	**Day 7**	**Day 0**	
138.75±9	111.25±6.4	92.5±6.5	65.63±6.5	48.75±3.5	**IL-2 (pgr/mL)**
8.54±178.75	10.28±138.13	8.51±91.88	5.20±36.25	1.44±3.75	**IL-4 (pgr/mL)**
1.65±93	4.42±33.31	1.61±17.06	1.39±6.91	0.60±2.84	**IL-12 (pgr/mL)**
76.29±1.42	57.75±1.08	40.46±3.99	20.88±1.42	10.67±0.96	**IFN-γ** ** (pgr/mL)**
34.57±3.89	22.83±1.02	19.78±0.64	9.35±0.47	1.20±0.42	**IgM** ** (pgr/mL)**
50.510.93	20.20±0.73	7.05±0.25	3.96±0.25	1.34±0.54	**IgG** ** (** **μ** **gr/mL)**
**pBudCE4.1**					
100±3.2	87.5±4.3	75±1.4	65.625±2.04	48.75±3.5	**IL-2 (pgr/mL)**
58.1±3.1	38.75±4.3	26.8±2.3	15.62±3.75	3.75±1.44	**IL-4 (pgr/mL)**
66.59±6.5	30.96±2.18	24.87±1.3	18.31±1.61	2.84±0.59	**IL-12 (pgr/mL)**
51.29±9.1810	36.7±2.08	26.08±2.09	19.4±1.73	6±096	**IFN-γ** ** (pgr/mL)**
27.22±0.54	23.31±0.76	20.9±0.94	14.23±0.74	1.19±0.4	**IgM** ** (pgr/mL)**
14.6±1.2	9.8±0.5	7.4±1.02	4.4±0.5	1.3±0.5	**IgG** ** (** **μ** **gr/mL)**
**PBS**					
87±4.7	80.625±2.3	73.125±3	65.62±3.7	48.75±3.5	**IL-2 (pgr/mL)**
36.25±3.22	26.25±3.2	16.8±4.2	6.8±2.3	3.1±1.25	**IL-4 (pgr/mL)**
33.6±4.9	25.5±0.5	19.4±2.24	9.09±1.38	2.84±0.59	**IL-12 (pgr/mL)**
34.6±2.03	27.95±2.08	24.20±1.04	16.5±1.5	10.6±096	**IFN-γ** ** (pgr/mL)**
13.8±0.9	12.9±6.61	7.01±0.48	4.56±0.53	1.19±0.41	**IgM** ** (pgr/mL)**
10.5±0.8	6.13±0.33	4.92±0.46	3.20±0.57	1.3±0.5	**IgG** ** (** **μ** **gr/mL)**

Data presented as Mean±SD. The *, **, ***, and **** indicate *P*-value less than 0.05, 0.01, 0.001, and 0.0001, respectively

Data presented as Mean±SD. The *, **, *** and **** indicate the *P*-value less than 0.05, 0.01, 0.001 and 0.0001 respectively

## Discussion

According to the Maastricht consensus, the treatment efficiency of **a** standard triple therapy of *H. pylori* based on bismuth, proton pump inhibitor (PPI), tetracycline, and metronidazole has recently decreased to undesirable levels in most countries (24, 25). Several factors challenge the *H. pylori*-associated infection treatment, including development of antibiotic-resistant strains of *H. pylori*, inoculum effect, genetic diversity of *H. pylori* during the infection cycle, and protection of bacteria via the thick gastric mucus gel layer(24). These factors may make *H. pylori* become inaccessible to antibiotics (24, 25). In the case of *H. pylori*-resistant strains, a meta-analysis of 87 studies from 2009- 2014 by Debraekeleer *et al*. revealed average rates of *H. pylori* antibiotic resistance 47.22% for metronidazole, 19.74% for clarithromycin, 18.94% for levofloxacin, 14.67% for amoxicillin, 11.70% for tetracycline, 11.5% for furazolidone, and 6.75% for rifabutin (26). Meanwhile, Africa and Oceania have been reported as regions with the highest and lowest prevalence of *H. pylori, *respectively (4). Australasia, Switzerland, more generally, North America, and Western Europe have the least incidence of *H. pylori* infection (27). In addition to the efficacy of treatment, there were also several disadvantages connected to the antibiotic-based approach such as intestinal discomfort, infection with opportunistic pathogens such as *Clostridium difficile*, inflammatory bowel syndromes, metabolic diseases like obesity, diabetes, and fatty liver disease (28). This is not the end of the story, and World Health Organization (WHO) has classified *H. pylori* as a class I human carcinogen which is involved in gastric cancer progression (29). When the drawbacks mentioned above are considered, looking for a new alternative to the treatment of *H. pylori *infections becomes urgent (26). There would be a great benefit to society if safe, effective, and low-cost vaccines were available to prevent chronic *H. pylori* infection (30). In the past decades, DNA vaccine strategy has risen to prominence in biology as a tool for acquiring immunogenicity against human pathogens like *H. pylori *(31). Hundreds of publications have introduced a variety of *H. pylori* antigens to develop DNA vaccines. Besides, DNA vaccine seems to work as a suitable approach for non-culturable bacteria or those similar to *H. pylori, *whose culture in liquid media, is difficult and needs nutrient-rich media (32). DNA vaccines can also suppress the unwanted effects of lengthy treatments using broad-spectrum antibiotics. DNA vaccines can induce both cellular and humoral immune responses in animals. Some DNA vaccines already have been licensed for infectious diseases (33). Such vaccines are very stable at room temperature, the feature that facilitates their transport and storage (34). In the present study, we have benefited from the pBudCE4.1 eukaryotic co-expression vector for transferring the *H. pylori*-associated *flaA* antigen into the eukaryotic host cells. The *Flagellin A* gene was considered as its encoded protein as a critical factor in the initial colonization of *H. pylori* and to attain robust infection (35). Looking through literature it can be found that the potential of colonization of *flaA*^-^ mutant is 10^-4^ times less than wild-type counterparts (36). Using an *in silico* analysis, Zarei showed that flaA protein is a potent immunogenic factor in *H. pylori* (37). It is important to note that bacterial flagellin such as *flaA* has adjuvant and immunomodulatory potential and activates immune and non-immune cells through the germline-encoded pattern recognition receptor TLR5 (38). Bacterial flagellin can stimulate both native and adaptive immunity(39). This is the reason that no adjutant agents were used in this study. Flagellin-adjuvanted vaccines show effective mucosal adaptive immune responses to both flagellin and co-administrated antigen. This due to the existence of TLR5 on epithelial cells, which are usually the first major cell types to meet the infectious agents, suggests flagellin as a mucosal adjuvant (38, 39). Until now, flagellin has been widely used as a mucosal adjuvant against epitope-based influenza vaccines, *West Nile virus* (WNV),* Escherichia coli*, *Yersinia pestis*, *Clostridium tetani*, *C. jejuni* *Streptococcus, *and *Plasmodium falciparum *(38). The use of flagellin as an adjuvant has produced safe, potent vaccines, and some of these vaccines reached human clinical trials (38). Our study was undertaken as the used cloning vector contains two strong viral promoters P_CMV_ and P_EF-1α _that make it suitable for simultaneous expression of two genes in mammalian cell lines and constructing multi-gene plasmid. We have firstly cloned *flaA* into the pTZ57RT vector and subsequently sub-cloned it into pBudCE4.1. Both processes were validated using DNA sequencing and double digestion. Then recombinant pBudCE4.1*-flaA* was successfully introduced into the HDF cells and expressed the recombinant protein as expected. Following the IM injection of recombinant construct in the experimental mice model, we found that the serum levels of INF-γ, IgM, IgG, IL-2, IL-4, and IL-12 were significantly increased in a time-dependent manner. We observed that the serum levels of IL-4, IL-2, IFN-γ, and IL-12 were increased at day 35 in mice who received the pBudCE4.1-*flaA* recombinant construct. Th_1 _cell-secreted IL-2 and IFN-γ mediated cellular immunity. Similarly, Lindholm showed that IFN-γ, IL-1, IL-6, and IL-8, but not IL-4 were increased in the *H. pylori*-infected persons compared with the levels in the healthy individuals (40). It has been shown that IL-12 is a dendritic cell-producing cytokine that is actively involved in the differentiation of T helper cells toward the Th_1 _subset (41)_. _As stated, IL-12 was one of the cytokines induced following the injection of our recombinant construct. However, IL-4 is produced by Th_2_ and involved in B-cell antibody secretion and down-regulation of chronic inflammatory reactions (42). IL-2 was the prominent cytokine in the serum following the first days of injection. This finding was inconsistent with the previous studies suggesting the Th_1_ response as the dominant response in *H. pylori*-infected mucosa (41). In a study, IL-2 was reported as a T cell growth factor which increased the response to *H. pylori* LPS (43). However, scientists are in doubt that Th_1_-associated immunity is a protective response or if it is associated with the pathogenesis of *H. pylori*s’ diseases (40). We also found that IgM is the first detected antibody in serum of mice receiving recombinant vectors showing the active response against *flaA* containing construct. This finding was in agreement with the observation by Nurgalieva (44). In parallel with IgM, the increased level of IgG was also observed in a time-dependent route and at day 35 of post-injection passed the IgM level. The increased level of IgG was previously reported in a variety of studies on *H. pylori*-infected individuals (45). Until now, numerous experimental tactics to mediate protection against *H. pylori* infection have been done. These included the whole lysate, inactivated strain of H. pylori and recombinant proteins, but when we looked at the status of vaccine research and development for *H. pylori*, we observed there were no effective vaccine candidates with only a single vaccine (Imevax/IMX101) in Phase I clinical trial (46). IMX101 has been designed based on an outer membrane protein of *H. pylori,* γ-glutamyltranspeptidase (GGT) as antigen, and a mucosal adjuvant. It seems that GGT has quite potent immunosuppressive activity (47). Some protection was observed by other vaccine candidates such as EpiVax, Helicovaxor, Urease epitope vaccine, and p220 vaccine; however, all of them are in the preclinical phase (46). Vaccine failures are mostly due to immune evasion by this pathogen (48). There are also other reasons for such failure: antigen which is selected for vaccine development should be shared by all *H. pylori* isolates without intrinsic toxicity (49). As an example, cytotoxin VacA is not expressed by all strains of *H. pylori *and may not be an ideal vaccine candidate (50). Antigen-like heat shock protein HspB has also been suggested, while this protein has homologies to the GroEL family of heat shock proteins which play a critical role in autoimmune reactions (51). Urease and HspA are expressed by all *H. pylori* isolates and were not connected to side effects when administered orally with an adjuvant to mice (52). This information shows that there is a long way to develop an effective vaccine against *H. pylori*, and continued research on this topic can complete the vaccine puzzle for this pathogen. Meanwhile, DNA vaccines are promising. 

## Conclusion

The findings of current research showed that the pBudCE4.1-*flaA* construct was able to activate the immune responses. This study is the first step towards the synthesis of recombinant-construct based on the *flaA *gene. Immunization with such construct may inhibit the *H. pylori*-associated infection; however, further experiments are urgent.
